# Cloning and sequencing analysis of whole *Spiroplasma* genome in yeast

**DOI:** 10.3389/fmicb.2024.1411609

**Published:** 2024-05-31

**Authors:** Masaki Mizutani, Sawako Omori, Noriko Yamane, Yo Suzuki, John I. Glass, Ray-Yuan Chuang, Takema Fukatsu, Shigeyuki Kakizawa

**Affiliations:** ^1^Bioproduction Research Institute, National Institute of Advanced Industrial Science and Technology (AIST), Tsukuba, Japan; ^2^Synthetic Biology Group, J. Craig Venter Institute, La Jolla, CA, United States; ^3^Telesis Bio, San Diego, CA, United States; ^4^Department of Biological Sciences, Graduate School of Science, The University of Tokyo, Tokyo, Japan; ^5^Graduate School of Life and Environmental Sciences, University of Tsukuba, Tsukuba, Japan

**Keywords:** *Spiroplasma*, whole genome cloning, synthetic biology, yeast artificial chromosome vector, transformation-associated recombination (TAR) cloning

## Abstract

Cloning and transfer of long-stranded DNA in the size of a bacterial whole genome has become possible by recent advancements in synthetic biology. For the whole genome cloning and whole genome transplantation, bacteria with small genomes have been mainly used, such as mycoplasmas and related species. The key benefits of whole genome cloning include the effective maintenance and preservation of an organism's complete genome within a yeast host, the capability to modify these genome sequences through yeast-based genetic engineering systems, and the subsequent use of these cloned genomes for further experiments. This approach provides a versatile platform for in-depth genomic studies and applications in synthetic biology. Here, we cloned an entire genome of an insect-associated bacterium, *Spiroplasma chrysopicola*, in yeast. The 1.12 Mbp whole genome was successfully cloned in yeast, and sequences of several clones were confirmed by Illumina sequencing. The cloning efficiency was high, and the clones contained only a few mutations, averaging 1.2 nucleotides per clone with a mutation rate of 4 × 10^−6^. The cloned genomes could be distributed and used for further research. This study serves as an initial step in the synthetic biology approach to *Spiroplasma*.

## Introduction

The synthetic biology has expanded greatly in recent years, making it possible to handle long DNA and conduct research using it (Kelwick et al., 2014; Venter et al., 2022). Bacteria with small genomes, such as *Mycoplasma* species and allied bacteria, have been used as targets in synthetic biology. Several studies have reported that their entire genomes were synthesized, cloned, modified, and transplanted into other bacterial cells. For example, whole genome cloning of several bacteria were reported including *Mycoplasma genitalium* (Gibson et al., 2008), *Mycoplasma mycoides* (Benders et al., 2010; Gibson et al., 2010)*, Mycoplasma capricolum* and related species (Labroussaa et al., 2016), *Acholeplasma laidlawii* (Karas et al., 2012), *Synechococcus elongatus* PCC 7942 (Noskov et al., 2012), *Prochlorococcus marinus* MED4 (Tagwerker et al., 2012), and *Mesoplasma florum* (Baby et al., 2018). The entire genome of *M. mycoides* and its related species were transplanted into *M. capricolum* recipient cells (Lartigue et al., 2007, 2009; Labroussaa et al., 2016; Baby et al., 2018). The entire genome of *M. mycoides* was chemically synthesized, cloned into yeast, and transplanted into *M. capricolum* recipient cell to create JCVI-syn1.0, a bacterium with a chemically synthesized genome (Gibson et al., 2010). Based on JCVI-syn1.0, a minimal cell JCVI-syn3.0, whose genome retains only essential genes, was constructed (Hutchison et al., 2016). Techniques for editing the entire genome of bacteria cloned into yeast have also been reported (Chandran et al., 2014; Tsarmpopoulos et al., 2016; Zhao et al., 2023). The advantages of enabling whole genome cloning encompass the ability to preserve the entire genome of an organism, the possibility of genome modification using the genetic engineering system in yeast, and its subsequent use in further research.

Members of the genus *Spiroplasma* comprise a bacterial group closely related to *Mycoplasma* (Davis et al., 1972). *Spiroplasma* species, characterized by their spiral cell shape and rotational swimming motility, infect a wide range of hosts including plants, insects, crustaceans, and mammals (Whitcomb, 1980; Cisak et al., 2015). *Spiroplasma citri* and *Spiroplasma kunkelii* are notorious to cause significant damage to citrus and corn, respectively (Whitcomb et al., 1986). *Spiroplasma mirum* is reported to cause cataracts and neurological damage in suckling mice (Tully et al., 1982). *Spiroplasma* species are detected in a wide variety of insects (Regassa and Gasparich, 2006; Cisak et al., 2015; Kakizawa et al., 2022), some of which cause male-killing phenotypes in fruit flies and other insects. In *Drosophila* fruit flies, *Spiroplasma poulsonii* induces male-killing, wherein an effector protein named Spaid was identified to induce male-specific apoptosis during embryogenesis (Harumoto and Lemaitre, 2018; Harumoto, 2023). Thus far, many *Spiroplasma* species remain unculturable, making it extremely difficult to elucidate their detailed characteristics. Furthermore, the absence of genetic knockdown or overexpression systems in most *Spiroplasma* strains has impeded experimental studies on their functional aspects. Recently, however, this difficulty was overcome, at least partially, by adoption of heterologous expression system in *Mycoplasma* cells. When cytoskeletal genes *mreB*s of *Spiroplasma* were expressed in *Mycoplasma* cells, the transformed *Mycoplasma* cells showed spiral cell shape and swimming motility (Kiyama et al., 2022; Lartigue et al., 2022). Such a new approach to investigate the genetic and functional aspects of *Spiroplasma* is anticipated.

In this study, we report cloning of the whole genome of *Spiroplasma chrysopicola* isolated from a deer fly *Chrysops* sp. (Diptera: Tabanidae) (Whitcomb et al., 1997; Ku et al., 2013) and the whole genome resequencing of the obtained clones. Although the size of the entire genome as large as 1.12 Mbp, the cloning efficiency was high, and the cloned sequences contained only a small number of mutations (1.2 nucleotides per clone), confirming that the whole *Spiroplasma* genome can be cloned in yeast cells with little alteration to the original genome information. This study serves as an initial step in the synthetic biology approach to *Spiroplasma*.

## Materials and methods

### *Spiroplasma* and yeast strains, cultivation, and culture media

The bacterial strain *S. chrysopicola* DF-1 was obtained from the American Type Culture Collection (ATCC 43209), which was cultured statically at 30°C using SP-4 medium (Tully et al., 1979). The cell growth was judged by the color of phenol red, a pH indicator. Cell morphology of *S. chrysopicola* was observed by optical microscope (IX71; Olympus). The yeast strain *Saccharomyces cerevisiae* VL6-48 was obtained from the ATCC (ATCC MYA-3666), which was cultured at 30°C using YPDA medium or SD-His medium (synthetic defined media lacking histidine, Clontech 630312) (Noskov et al., 2010).

### Preparation of genomic *Spiroplasma* DNA for TAR cloning

Preparation of circular genomic DNA from *S. chrysopicola* cells was performed according to a previously reported method (Lartigue et al., 2007). Briefly, *S. chrysopicola* cells were cultured, collected by centrifugation, suspended in a buffer (10 mM Tris-HCl, pH 6.5, 500 mM sucrose, 50 mM EDTA), mixed with an equal volume of 2% UltraPure low melting point agarose (Invitrogen), and poured into plug molds (BioRad) to prepare agarose gel plugs. The cells were digested for 2 days using 1 mg/ml Proteinase K and 1% SDS solution at 55°C, thoroughly washed with a washing buffer (20 mM Tris-HCl, pH 8.0, 50 mM EDTA), and stored at 4°C. The circular genomes within the plugs were digested with restriction enzymes *Asc*I, *Sfi*I, and I-CeuI (New England Biolabs), and then the agar plugs were melted using thermostable beta-agarase (Nippon Gene). Pulsed-field gel electrophoresis (PFGE) was performed as described (Gibson et al., 2010). The condition of electrophoresis was 6 V, 50–90 sec pulse time for 22 h at 14°C. DNA bands were visualized with GelRed (Biotium Inc.).

### Preparation of yeast artificial chromosome cloning vector

A summary of the plasmid YAC vector construction is shown in Supplementary Figure S1. PCR was performed using pRS313 plasmid as a template and primers (ROC800 and ROC801) to amplify a 4.2 kbp fragment containing *his3* gene, a selection marker gene in yeast. PCR was performed using the *M. mycoides* JCVI-syn1.0 genome as a template and primers (ROC802 and ROC803) to amplify a 6.2 kbp fragment containing yeast centromere. These PCR products were purified using PCR purification kit (QIAquick PCR Purification Kit; Qiagen), introduced into yeast VL6-48 cells, and assembled by *in vivo* homologous recombination to create pRC65 vector (10,415 bp). The pRC65 plasmid could multiply both in yeast and *Escherichia coli*, and be used as YAC cloning vector. The pRC65 plasmid was extracted from the yeast cells using Miniprep kit (QIAprep Spin Miniprep Kit; Qiagen) and introduced into *E. coli* DH5-alpha cells. The plasmid samples were extracted from the *E. coli* cells using Miniprep kit (Qiagen) and then sequenced by the Sanger sequencing method. PCR was performed using the pRC65 as a template and SChry_TAR primers to obtain a 6.8 kbp PCR product. These primers include flanking sequences homologous to the terminal regions of each genomic fragment within the *S. chrysopicola* genome. The PCR products were purified using PCR purification kit (Qiagen) and used as vectors for the whole genome cloning. Primer sequences are shown in the Supplementary Table S1.

### Primer design for colony PCRs

Primers for colony PCR to confirm the inserts were designed based on single-copy genes in the *S. chrysopicola* genome (Ku et al., 2013) to ensure specific amplification of certain genomic locations. To detect single-copy genes, BLAST analysis was performed on the *S. chrysopicola* genome using all *S. chrysopicola* genes as queries, and genes for which only one homolog was detected were designated as single-copy genes. Among detected single-copy genes, those that were evenly distributed across the genome were selected. In total 28 primer sets were designed on the genome, with seven sets for each genomic fragment. The positions of the designed primers and names of selected single-copy genes are listed in the Supplementary Figure S2, and sequences of the designed primers are shown in Supplementary Table S1.

### Transformation-associated recombination cloning

The cloning method followed previously reported methods (Lartigue et al., 2009; Benders et al., 2010; Kouprina and Larionov, 2016). The circular S. *chrysopicola* genome was digested with the same restriction enzymes, and a mixture of 100 ng of the vector fragment and 1 μg of digested *S. chrysopicola* genome was used for yeast transformation.

In brief, the yeast strain was cultured in 50 ml of YPDA medium at 30°C with shaking until the OD_600_ reached 6.0–7.0. After harvesting by centrifugation (1,900 × g, 4°C, 5 min), the cells were suspended in a 1 M sorbitol solution and incubated at 4°C overnight. The cells were then treated with the cell wall lytic enzyme Zymolyase (NACALAI TESQUE) to prepare spheroplasts in a phosphate buffer (pH 7.5) containing 1 M sorbitol, 10 mM EDTA, and 0.2% (v/v) beta-mercaptoethanol. After washing with the 1 M sorbitol solution, the cells were resuspended in STC buffer (1 M sorbitol, 10 mM Tris-HCl, pH 7.5, 10 mM CaCl_2_) and incubated at room temperature for 10 min. The cells were then mixed with vector and genomic DNA inserts and incubated at room temperature for 10 min. A 20% polyethylene glycol (PEG) 8000 solution was added, mixed gently, and incubated at room temperature for 20 min. The cells were collected by centrifugation (3,200 × g, 5 min), resuspended in SOS solution (1 M sorbitol, 6 mM CaCl_2_, 0.3% yeast extract, 0.6% peptone), and incubated at 30°C for 30 min. Finally, the cells were mixed with the heat-melted SD-His TOP agar medium containing 1 M sorbitol, 2% glucose, and 3% agar, then spread onto SD-His agar plates. Selection of transformed yeast strains was performed on SD-His medium.

### Analysis of YAC clones

To visualize insert bands in YAC clones, combination of conventional agarose gel electrophoresis and PFGE were performed. To exclude yeast chromosomes, conventional agarose gel electrophoresis was performed. The yeast clones containing the *S. chrysopicola* genome were cultured in SD-His medium until the OD_600_ reached 1.0 – 1.5 and embedded in agarose plugs. After treatment of the agarose plugs with Zymolyase, Proteinase K, and SDS for 2 days at 55°C, linear yeast chromosomes were flushed out from the agarose plugs by conventional electrophoresis (1% agarose gel, 100 V for 3 h at 4°C). This method is based on the phenomenon that only linear DNA migrates from the agarose plugs during electrophoresis, while circular, large DNA persists in the agarose plugs (Lartigue et al., 2009). After electrophoresis, agarose plugs were picked up from the agarose gel, washed with the washing buffer, and treated with *Not*I restriction enzyme for 2 h at 37°C. Since the cloning vector has two *Not*I recognition sites (Supplementary Table S1), the circular YAC vector including the genome insert was digested between the vector-insert boundaries. Then, the agarose plugs were washed with the washing buffer, and subjected to PFGE under the conditions of 6 V, 50–90 sec pulse time for 22 h at 14°C. DNA bands were visualized with GelRed.

The yeast clones containing the *S. chrysopicola* genome were cultured and embedded in agarose plugs, and liner DNAs were removed as described above. Then, from the agarose plugs, remaining circular DNA consisting of YAC including the *S. chrysopicola* genome was extracted using NucleoSpin Tissue kit (Macherey-Nagel). The extracted DNA was amplified using phi29 DNA polymerase (Thermo Scientific) and subjected to Illumina sequencing. The sequence reads were mapped onto the original genome sequence (GenBank accession number NC_021280.1), then mutations in cloned genomes were detected using CLC Genomics Workbench (Filgen).

## Results

### Preparation of whole *Spiroplasma* genome

**Figure 1:**
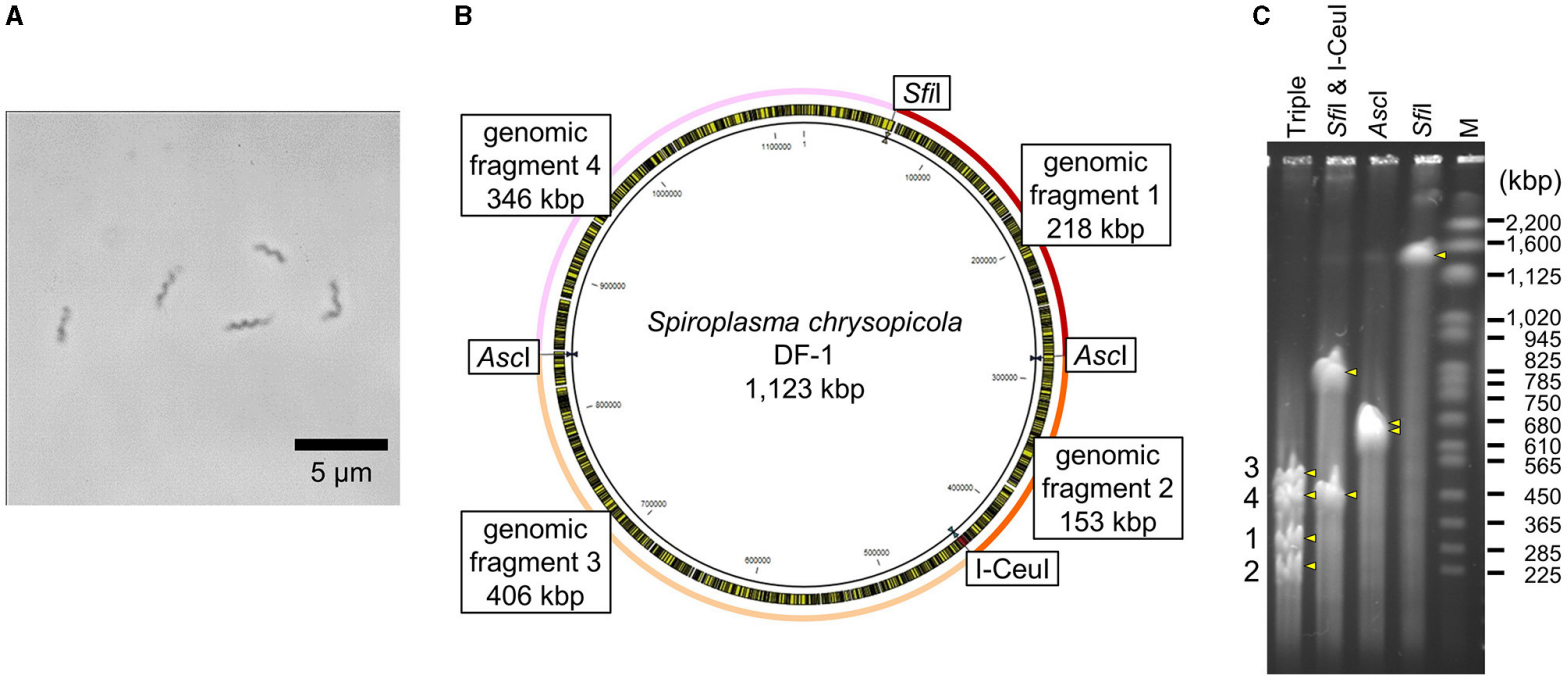
Genomic features of *S. chrysopicola*. **(A)** Spiral cell morphology of *S. chrysopicola*. **(B)** Genome map of *S. chrysopicola* DF-1 strain (1,123 kbp). Recognition sites for restriction enzymes *Asc*I, *Sfi*I and I-CeuI used, and four genome fragments produced by digestion with the enzymes are shown. **(C)** PFGE profiles of the *S. chrysopicola* genome after digestion with the restriction enzymes. Bands are indicated by yellow arrowheads. Four genome fragments, which correspond to those shown in **(B)**, are labeled on the left. Lane labels: Triple, triple digestion with *Asc*I, *Sfi*I and I-CeuI; *Sfi*I & I-CeuI, double digestion with *Sfi*I and I-CeuI; *Asc*I, single digestion with *Asc*I; *Sfi*I, single digestion with *Sfi*I; M, *Saccharomyces cerevisiae* chromosome marker (BioRad). Sizes of marker bands are shown on the right.

**Figure 2:**
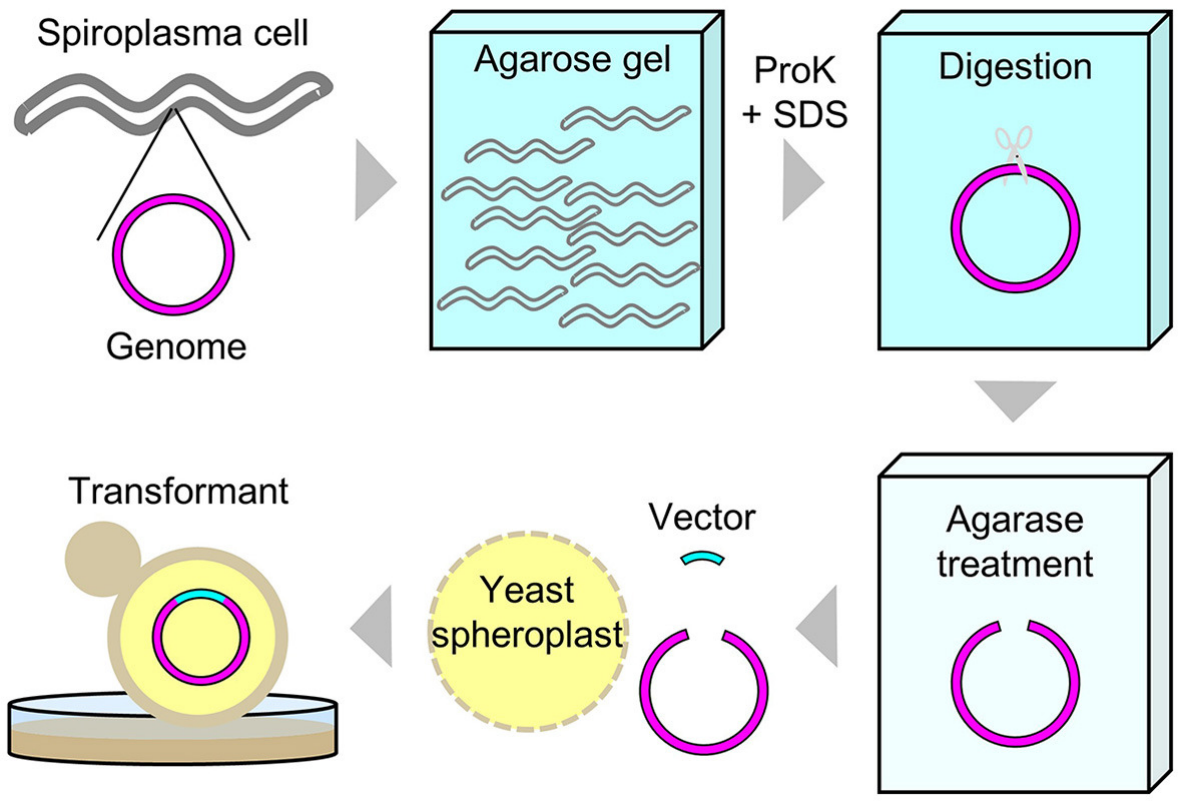
Schematic diagram of experimental procedures for the whole genome cloning of *S. chrysopicola*.

Intact circular genomic DNA of *S. chrysopicola* was prepared from cultured bacterial cells embedded in agarose plugs. The agarose gel plugs were digested with restriction enzymes *Asc*I, *Sfi*I, and I-CeuI, and then subjected to PFGE. Length of all the fragments cut by the enzymes *Asc*I, *Sfi*I, and I-CeuI were similar with the expected band patterns (Figure 1). Note that it has been estimated that PFGE has an uncertainty in size determination ranging from 5% to 27% (Huang et al., 1999; Duck et al., 2003; Ferris Matthew et al., 2004). These digested fragments were used for further cloning experiments. By digestion with three enzymes (*Asc*I, *Sfi*I, and I-CeuI), four fragments were generated, all of which were used for genome cloning. Additionally, two types of whole genomes, each digested with either *Sfi*I or I-CeuI, were also used for genome cloning (Supplementary Table S2). Figure 2 illustrates the entire process from the isolation of the *S. chrysopicola* genome to cloning.

### Preparation of cloning vector and whole genome cloning

**Figure 3:**
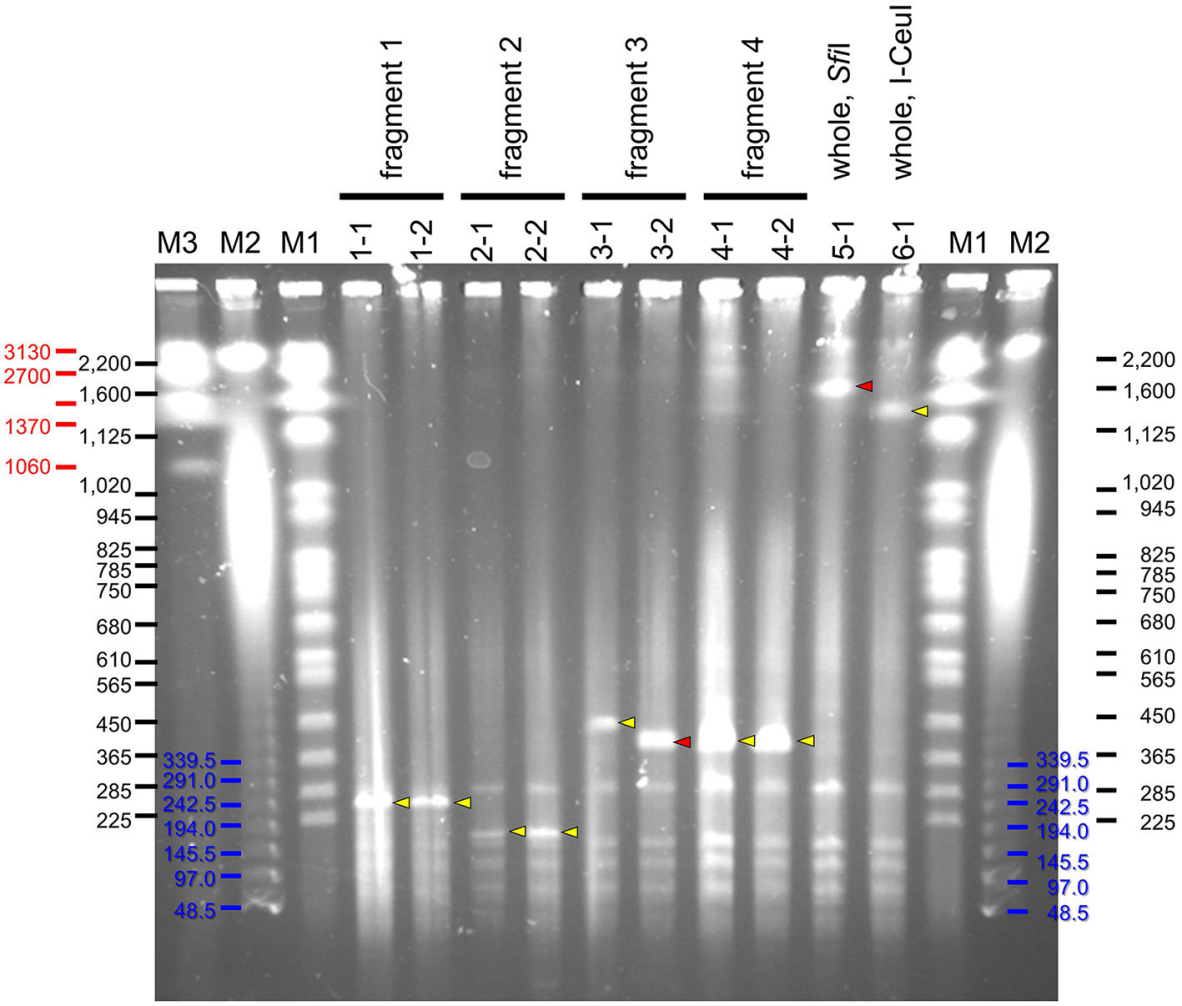
PFGE profiles of YAC clones inserted with genome fragments of *S. chrysopicola*. Yellow arrowheads indicate specific bands of expected size, whereas red arrowheads show specific bands of unexpected size. Lane labels: M1, *Saccharomyces cerevisiae* chromosome marker (BioRad), whose band sizes of bands are shown in black; M2, λ ladder marker (BioRad), whose band sizes are shown in blue; M3, *Hansenula wingei* chromosome marker (BioRad), whose band sizes are shown in red. For the other labels, see fragment numbers and clone numbers shown in Figure 1 and Supplementary Tables S1, S2.

Four partial genome fragments and two whole genome fragments of *S. chrysopicola* were used for cloning. As a result, a large number of transformed yeast colonies were obtained for all the genome fragments (Supplementary Table S2). Sixteen clones were isolated for each of the genome fragments, the presence of the insert was checked by colony PCRs, and positive clones were obtained for most of the genome fragments (Supplementary Table S2). It was observed that the smaller the insert size, the higher the proportion of positive clones. Particularly in the fragment No. 2 (153 kbp), 100% of the clones (16/16) were judged positive by colony PCRs. Subsequent PFGE showed that most clones exhibited the expected fragment size (Figure 3). To exclude yeast chromosomes, the agarose plugs were subjected to conventional agarose gel electrophoresis in advance, but the removal was not complete and the remnant chromosomal DNAs persisted as background. Notwithstanding this, the sizes of the inserted genome fragments were clearly recognizable on the PFGE gels (Figure 3).

Notably, for two clones (3-2 and 5-1), their insert sizes were different from the expected ones. Particularly for the clone 5-1 (whole genome cut with *Sfi*I), although some colony PCRs failed to yield expected products presumably due to the absence of some genomic regions, PFGE results suggested that the insert size was longer than expected (Figure 3). For the clone 3-2, the insert size was shorter than expected, suggesting that the insert sequence would be partially missing.

### Sequence analysis of clones

**Figure 4:**
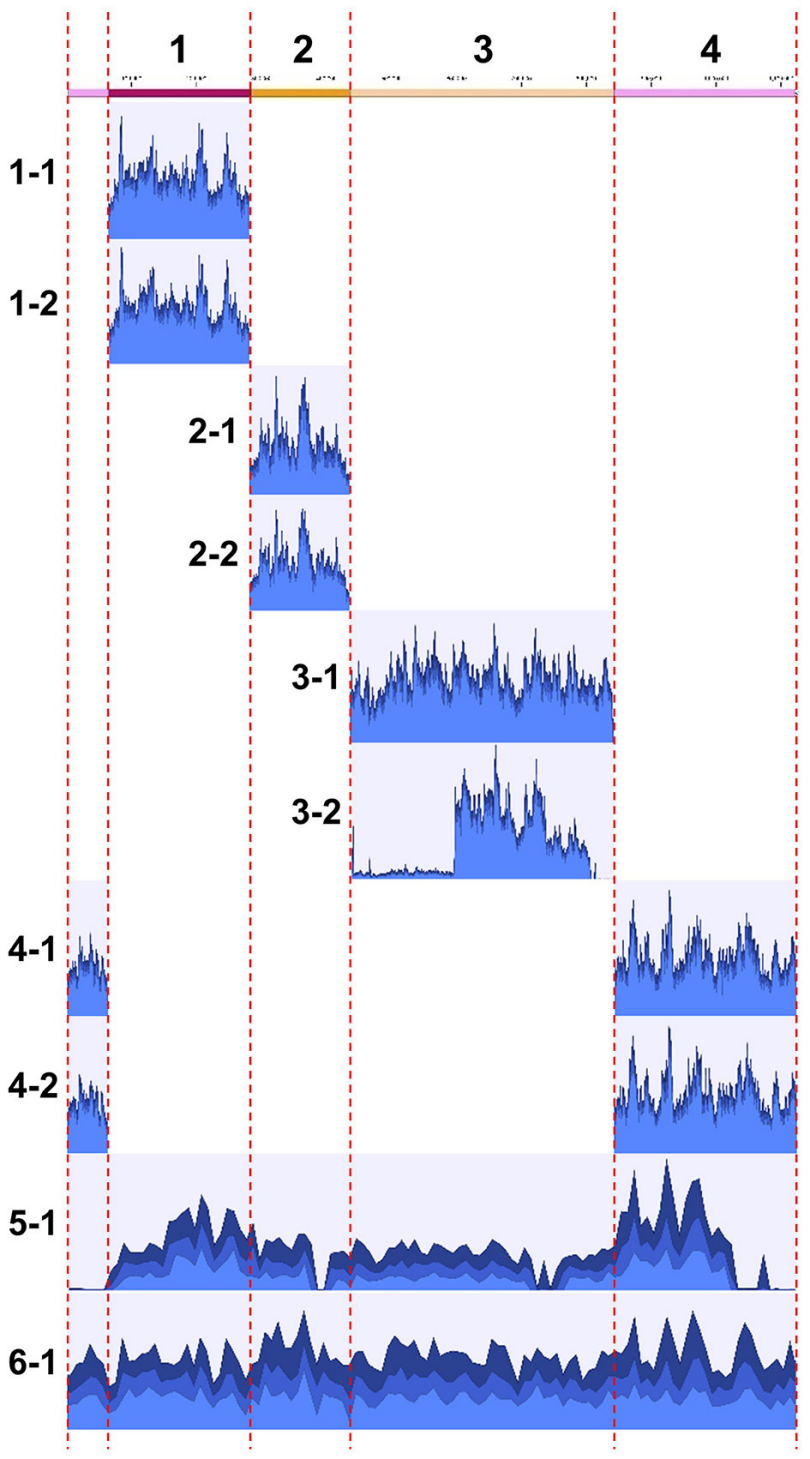
Mapping of Illumina short reads to the genome sequence of *S. chrysopicola*. On the left, clone numbers from Supplementary Table S2 are displayed. Note that the sizes of clones 3-2 and 5-1 are not consistent with the estimation by PFGE (see Figure 3).

The obtained clones were subjected to Illumina sequencing analysis. The same clones used for PFGE were also used for the sequencing analysis. After purification by PFGE, the DNA samples, which were expected to be inserted YAC clones, were subjected to whole genome amplification using phi29 DNA polymerase, and then to Illumina short read sequencing. For each of all the 10 clones analyzed, a sufficient number of reads were obtained and mapped onto the target *S. chrysopicola* genome (Figure 4; Supplementary Table S3). The proportion of sequence reads mapped onto the *S. chrysopicola* genome ranged from 2.9% to 36.4%, with an average of 18.8%. The remaining reads were mapped onto yeast chromosomes, mitochondrial DNA, and vector sequence. Coverage of the cloned inserts varied from 137 to 591-fold, with an average of 347-fold, indicating sufficient read quantity. Mapping results showed that most clones were covered by the mapped reads almost evenly, in general, across the entire length of the insert, as expected (Figure 4). For two clones (clone 3-2 and 5-1) whose insert sizes were estimated by PFGE as different from the expected sizes (Figure 3), resequencing results also confirmed differences in length from the reference sequence, indicating partial loss of the insert. The insert size of the clone 5-1 was estimated by PFGE as larger than expected (Figure 3), but resequencing results uncovered some missing regions, suggesting that the insert sequence might have partially duplicated during the cloning process.

Comparison with the reference genome revealed that an extremely low number of mutations, with 0 to 3 mutations observed in each clone, and some clones had no mutations at all (Supplementary Table S3). We judged them as mutations when more than 90% of the mapped reads did not match the reference genome but supported the mutation (>90% mapped frequency). Lowering this threshold to 30% detected some additional mutations (Supplementary Table S4), most of which were in regions with consecutive A or T bases, with an increase or decrease in the number of the consecutive bases. This might be due to the inaccuracy of the phi29 DNA polymerase on the consecutive bases.

The sum of the sizes of all clones (total number of bases cloned), excluding duplicated regions, was 2,963,696 bases, and the total number of mutations in these clones was 12 nucleotides, resulting in an average mutation rate of 4.0 nucleotides per Mb, highlighting highly accurate genome cloning.

## Discussion

### Cloning efficiency

We adopted the transformation-associated recombination (TAR) cloning method using the yeast *S. cerevisiae* as host cells (Kouprina and Larionov, 2016). The cloning efficiency observed in this study appears to be high. A large number of colonies were obtained with fewer experimental runs, and many clones contained inserts with the correct sequences. This might be related to the extent of repeat sequences in the insert genome fragments, since large parts of the genome could be lost through homologous recombination during TAR cloning procedures. It might also be related to the amount and concentration of the insert genome fragments (Rideau et al., 2017). It seems that the shorter the length of the insert, the higher the cloning efficiency, which is consistent with the characteristics of conventional cloning procedures. The cloning efficiency for inserts shorter than 200 kbp was exceptionally high, and cloning was possible even for inserts exceeding 1 Mbp. Circular YAC has been reported to clone inserts up to 1.66 Mbp (Tagwerker et al., 2012). Since many bacteria, archaea, and organelles with small genome sizes fall within this range, cloning their entire genomes seems feasible, in case that non-sheared, sufficient quantity of genomic DNA can be prepared.

### Sequence analysis of YAC clones

In this study, the YAC insert fragments after cloning were subjected to Illumina short read sequencing. In order to purify the inserted YAC vector DNA, we attempted to exclude linear yeast chromosomes from the agarose plugs by conventional agarose gel electrophoresis, whereby circular YAC DNA was expected to persist in the agarose plugs. Then, DNA was recovered from the agarose plugs, and subjected to phi29 polymerase amplification and Illumina sequencing. Unexpectedly, however, on average, only 18.8% of sequence reads were mapped onto the insert sequences, with the remaining reads mapped onto yeast chromosomes and mitochondrial DNA. Given that YAC behaves as one of the 16 chromosomes in yeast cells, theoretically, if total DNA was extracted and sequenced directly from yeast cells without any treatment, ~1% of the reads are expected to originate from the YAC sequence. Hence, the method adopted in this study could enrich YAC sequences by an average of 18-fold. Here it should be noted that, with the recent advancement in next-generation sequencing analysis, sufficient coverage for large DNA inserts can be achieved relatively easily by obtaining a large number of reads, even when insert ratio in the DNA sample is <1%.

### Mutation rate of clones

The resequencing of YAC clones revealed only a small number of mutations, and several clones were identical to the original genome sequence. Several single base deletions or insertions were observed in consecutive A or T bases, which were presumably introduced by amplification errors of phi29 DNA polymerase, but frequency of such mutations was low. In this study, circular whole genomes were extracted directly from cultured bacterial cells of *S. chrysopicola*, and theoretically, there are almost no steps where mutations are introduced into the bacterial genome, which may account for why the number of mutations in the inserts was at such a low level. A previous study reported the possibility of large degradation events in YAC-cloned large genome inserts after around 60 generations of yeast cultivation (Rideau et al., 2017). In this study, we used yeast cells cultured for 2-3 passages (12-18 generations) and many clones showed no mutations at all, indicating that the YAC-cloned large genome inserts are sufficiently stable at least in such a small number of yeast passages.

### Future prospects

The entire *Spiroplasma* genome cloned in this study could be utilized for a variety of future studies.

First, genetic modifications of the *Spiroplasma* genome using yeast genetic engineering tools are possible. In yeast, a variety of genetic engineering tools are available, e.g., CRISPR/Cas9 system (Tsarmpopoulos et al., 2016; Ruiz et al., 2019), TREC (Noskov et al., 2010), and TREC-In (Chandran et al., 2014).

Second, functional analysis through genome transplantation could also be possible. Thus far, successful whole genome transplantation has been reported only in a very limited number of *Mycoplasma* species in the *M. mycoides* group (Lartigue et al., 2007; Labroussaa et al., 2016). Certainly transplantation of the entire *Spiroplasma* genome must be challenging, but, considering the close phylogenetic relationship between *M. mycoides* group and *Spiroplasma* (Lo et al., 2013), it would become feasible in the future. To achieve this goal, various relevant factors should be examined systematically, including selection of recipient cells, modification of genome sequences, improvement of the transplantation methods, and verification and elimination of inhibitory effects of restriction enzymes or nucleases (Lartigue et al., 2009). For example, many bacteria, including *Spiroplasma* and *Mycoplasma* are known to possess restriction modification systems that confer resistance to phage invasion by cleaving foreign DNAs. The cloned genome in yeast is not methylated, therefore, it is likely to be digested when transplanted into bacterial cells. The efficiency of genome transplantation could be enhanced by using bacterial methylases to methylate the donor genome extracted from yeast cells (Lartigue et al., 2007), or by using recipient cells that lack nucleases (Labroussaa et al., 2023). These approaches might be also effective in *Spiroplasma* genome transplantation. If genome transplantation in *Spiroplasma* becomes possible, various genetic modifications would also be feasible, including the knockout or overexpression of certain genes, as well as the introduction of complete metabolic pathways or genetic systems. Furthermore, synthetic biology approaches could facilitate large-scale genomic deletions, insertions, or replacements. These techniques might enhance our understanding of *Spiroplasma* biology.

Third, our sequencing results showed a very limited number of mutations in the cloned *Spiroplasma* genomes, indicating that the genetic information of the cloned bacterial genome in yeast cells can be stably preserved with minimal alterations. This observation highlights the possibility that this technique could be potentially utilized as a tool for preserving and storing the whole undamaged microbial genomes. For example, preservation, storage, and usage upon necessity of such microbial genomes that are with extremely slow growth rates, requiring complex media or specific conditions for cultivation, or difficult to access, would be enabled by retaining their entire genomes within yeast cells. Yeast grows easily and rapidly, its culture medium is inexpensive and easy to prepare, it does not require specific facilities for cultivation, and yeast cells can be stably preserved in freezers for long periods. By making use of sophisticated genetic tools available for yeast, the whole microbial genomes cloned in yeast can be subjected to large-scale genetic manipulation of the original genomic DNA, allowing the cloned genomes to be utilized as genetic resources. Most of *Spiroplasma* isolates are cultured in SP-4 medium whose composition is complex and contains a considerable amount of expensive fetal bovine serum (Tully et al., 1979). Therefore, the yeast clones obtained in this study are considered useful for preparing large quantities of *Spiroplasma* genomic DNA. The whole-genome cloning technology is also expected to be useful for utilizing environmentally inaccessible microorganisms as genetic resources. We expect that, as the yeast-mediated bacterial whole-genome cloning technology becomes easier and more accessible, it will be applied to diverse microbial species and research purposes, thereby facilitating further utilization of the cloned microbial genomes.

## Data availability statement

The datasets presented in this study can be found in online repositories. The names of the repository/repositories and accession number(s) can be found at: https://www.ddbj.nig.ac.jp/, DRA018010-DRA018019.

## Author contributions

MM: Formal analysis, Investigation, Data curation, Writing—review & editing. SO: Investigation, Writing—review & editing. NY: Investigation, Writing—review & editing. YS: Conceptualization, Writing—review & editing. JG: Conceptualization, Writing—review & editing. R-YC: Conceptualization, Methodology, Writing—review & editing. TF: Project administration, Writing—original draft, Writing—review & editing. SK: Conceptualization, Formal analysis, Investigation, Data curation, Visualization, Project administration, Writing—original draft, Writing—review & editing.

## Supplementary material

Supplementary Figure S1 Schematic diagram of plasmid vector construction and whole genome cloning. Plasmid pRC65 was derived from pRS313 (available at Addgene) and a yeast centromere plasmid YCp. As a vector for whole genome cloning, regions homologous to the insert were added as flanking sequences of PCR primers. The schematic size of each gene does not correspond to its actual size.

Supplementary Figure S2 Genome map of *S. chrysopicola* with target genes of colony PCR. Seven single-copy genes from each of four genomic regions were selected for PCR amplification. Length of PCR fragments were designed to be differentiated by agarose gel electrophoresis.

Supplementary Table S1 List of primers used in this study.

Supplementary Table S2 Summary of whole genome cloning of *S. chrysopicola*.

Supplementary Table S3 Summary of resequencing of YAC clones.

Supplementary Table S4 Details of mutations in YAC clones.
