# Supplementary figures and images for: Cloning and sequencing analysis of whole Spiroplasma genome in yeast
Source: Front Microbiol. 2024 May 31;15:1411609. doi: 10.3389/fmicb.2024.1411609 (PMC11176537; doi:10.3389/fmicb.2024.1411609)

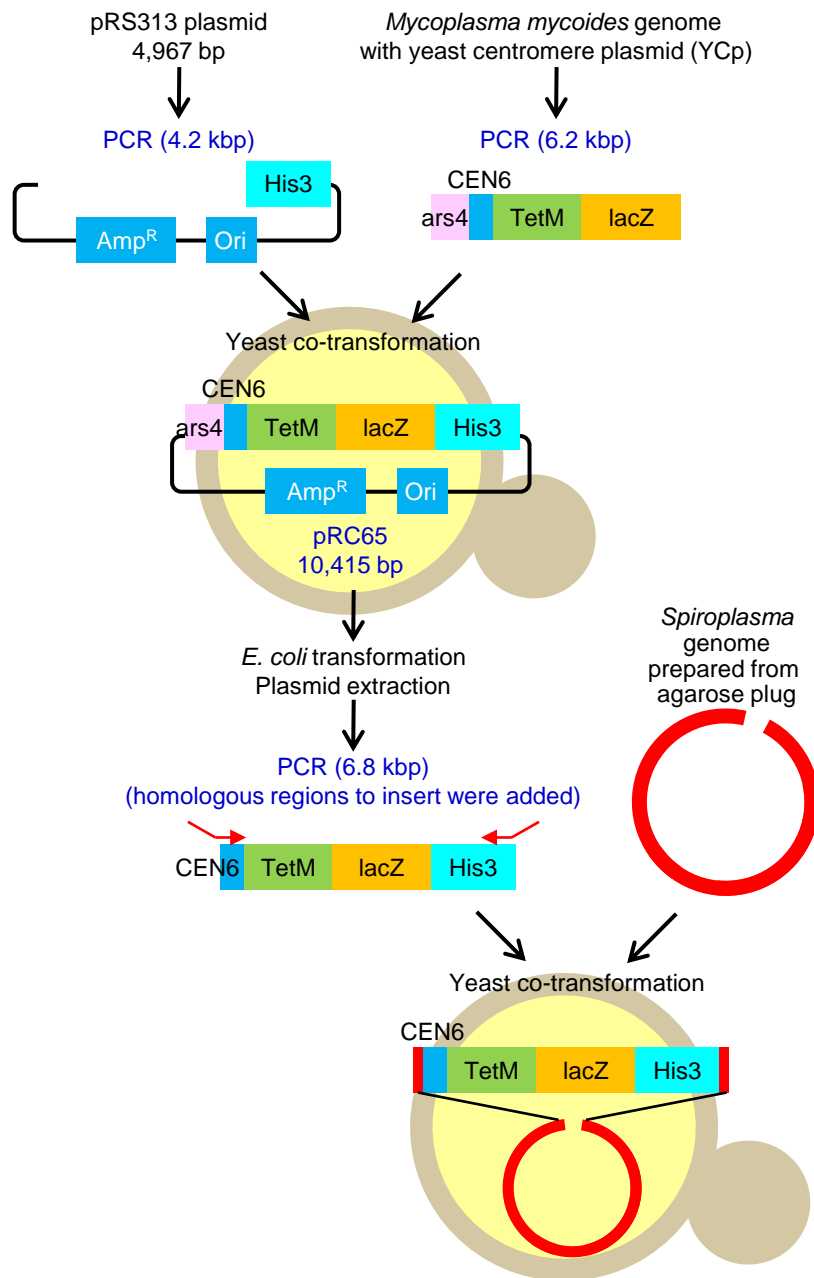

**SUPPLEMENTARY FIGURE S1**

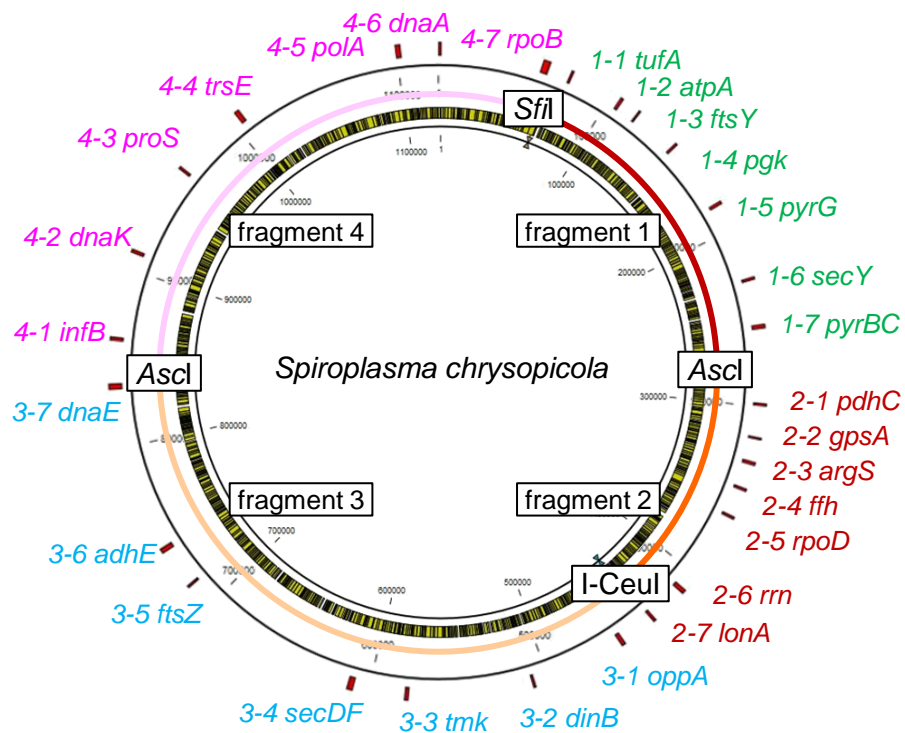

**SUPPLEMENTARY FIGURE S2**

Supplement: Supplementary Figure S1 — Schematic diagram of plasmid vector construction and whole genome cloning. Plasmid pRC65 was derived from pRS313 (available at Addgene) and a yeast centromere plasmid YCp. As a vector for whole genome cloning, regions homologous to the insert were added as flanking sequences of PCR primers. The schematic size of each gene does not correspond to its actual size. [file Data_Sheet_1.pdf]
